# Supplementary material for: Aging-associated decline of phosphatidylcholine synthesis is a malleable trigger of natural mitochondrial aging
Source: Nat Commun. 2026 Apr 18;17:3589. doi: 10.1038/s41467-026-71508-7 (PMC13091796; doi:10.1038/s41467-026-71508-7)
Supplement: Supplementary file 1 — Supplementary Information [file 41467_2026_71508_MOESM1_ESM.pdf]

Aging-associated decline of phosphatidylcholine synthesis is a malleable trigger of natural mitochondrial aging.

Tetiana Poliezhaieva, Yuting Li, Prerana Shrikant Chaudhari, Ulas Isildak, Pol Alonso Pernas, Isabela Santos Valentim, Fengting Su, Lilia Espada, Melike Bayar, Li Fu, Andreas Koeberle, Handan Melike Dönertaş and Maria A. Ermolaeva*.

*-Correspondence: Maria A. Ermolaeva (maria.ermolaeva@leibniz-fli.de). Leibniz Institute on Aging – Fritz Lipmann Institute (FLI), Beutenbergstrasse 11, 07745 Jena, Germany.

This file includes Supplementary Figure 1-16.

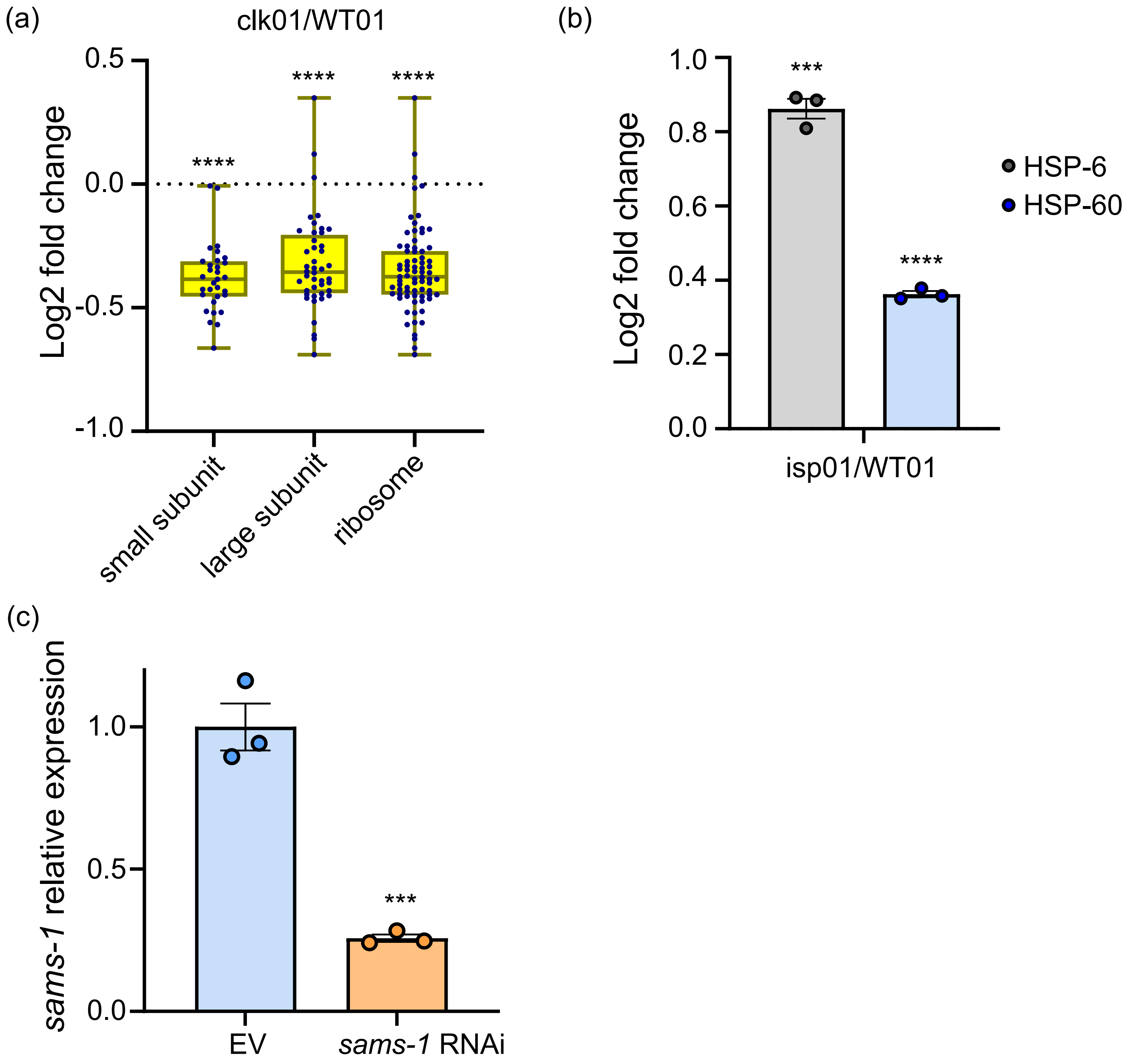

Supplementary Figure 1. Validation of proteomics results and *sams-1* gene knock down efficiency. (a–b) Proteomics samples were collected as described in Figure 1a. Box plots depicting relative expression (Log2 fold change) of all detected ribosomal proteins belonging to the small subunit, large subunit and whole ribosome between young (AD1) *clk-1(qm30)* and WT nematodes (labelled as clk01/WT01 comparison) are shown in **(a)**. Each dot represents one protein, median expression values are shown inside each box and whiskers highlight minimal and maximal values within each tested group. Statistics was assessed by Wilcoxon rank-sum test and all relevant expression values and calculations can be found in Supplementary Data 3. n=3 samples with 500 worms each were analyzed. **(b)** Relative expression values (Log2 fold change) of HSP-6 and HSP-60 mitochondrial chaperones between young (AD1) *isp-1(qm150)* and WT nematodes (labelled as isp01/WT01 comparison) are presented. Each dot represents one replica sample of 500 worms (n=3), mean and SEM values are presented and unpaired t-test was used to compute *p*-values. All relevant expression values and calculations can be found in Supplementary Data 4. **(c)** Quantitative RT-PCR analysis of *sams-1* mRNA levels in *sams-1* RNAi-treated worms, shown relative to control animals treated with empty vector (EV) RNAi; n=60 worms per sample, each dot corresponds to one technical replicate. In **c**, the experiment was repeated 3 times, and one representative result is shown, in **a**–**b** 3 independent biological replicas were measured for each condition. Mean and SEM values are presented in **b** and **c**; unpaired t-test was used for the statistical assessment. ***-$p<0.001$; ****-$p<0.0001$, all *p* values are two-tailed. Exact *p* values can be found in the Source Data file.

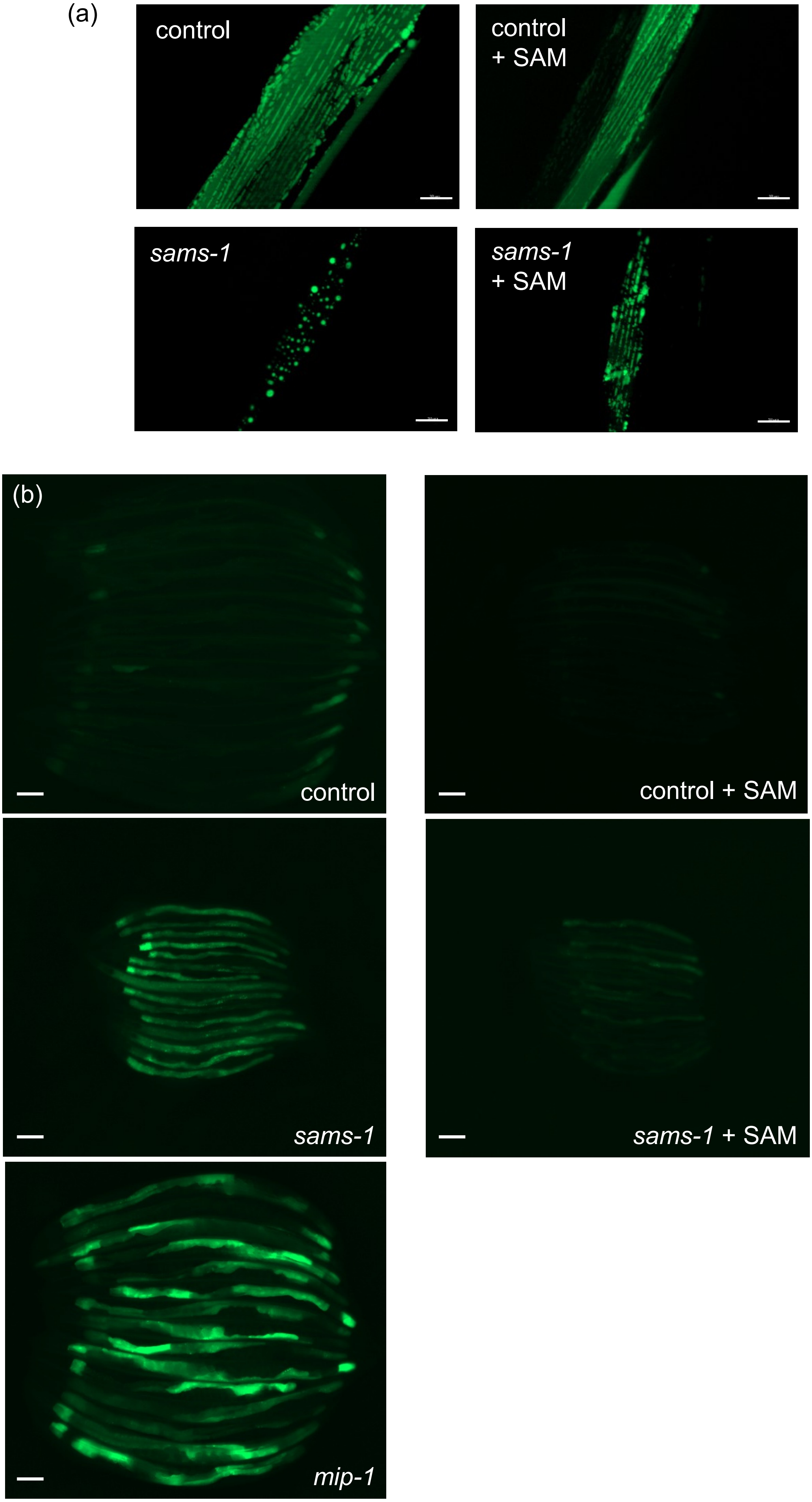

(a)
control
control
+ SAM
sams-1
sams-1
+ SAM
(b)
control
control + SAM
sams-1
sams-1 + SAM
mip-1

Supplementary Figure 2. Mitochondrial defects of *sams-1* KD animals are SAM-dependent. (a) Transgenic nematodes expressing GFP-tagged mitochondria in the body wall muscle *(myo-3p::gfpmit)* were age-synchronized and exposed to RNAi and compound treatment as in Figure 4b and representative mitochondrial morphology images are shown. Scale bar is 10µm. Images are from an independent replicate of the experiment quantified in Figure 4b. **(b)** Representative images of transgenic nematodes expressing GFP under the *hsp-6* promoter *(hsp-6p::GFP)* exposed to control and *sams-1* RNAi with or without 10mM SAM are shown, scale bar is 100µm. Images and treatments correspond to Figure 4c. Each experiment was repeated at least 3 times, and one representative result is shown in each case.

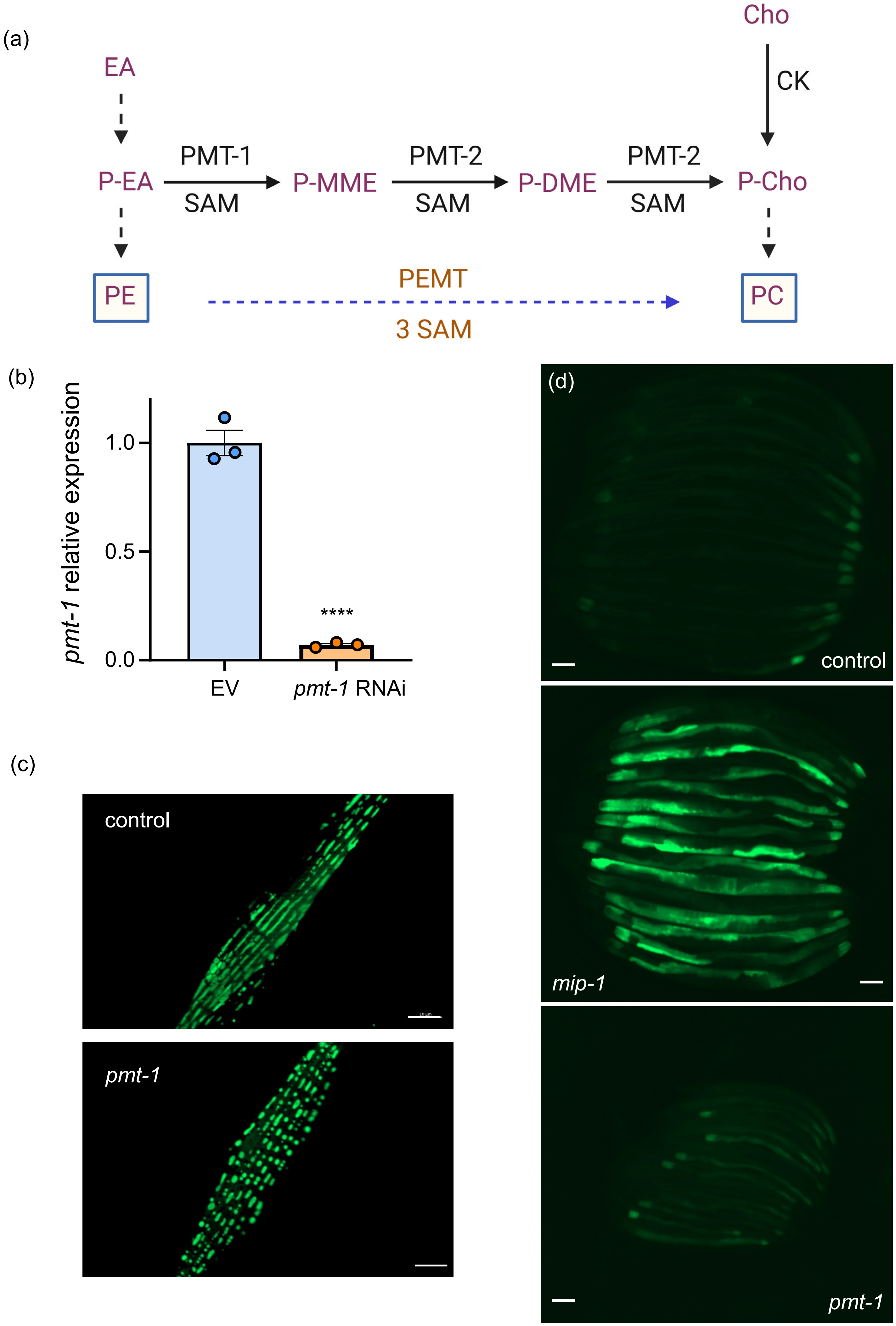

(a)
Cho
CK
EA
PMT-1
PMT-2
PMT-2
P-EA
P-MME
P-DME
P-Cho
SAM
SAM
SAM
PE
PEMT
3 SAM
PC
(b)
pmt-1 relative expression
1.0
0.5
0.0
****
EV
pmt-1 RNAi
(c)
control
pmt-1
(d)
control
mip-1
pmt-1

Supplementary Figure 3. *pmt-1* deficiency phenocopies mitochondrial morphology defects of the *sams-1* KD. (a) Schematic representation of relevant SAM-dependent (phospho-base methylation pathway, PEMT pathway) and independent (Kennedy pathway) mechanisms of phosphatidylcholine (PC) synthesis is shown. EA - ethanolamine, P-EA - phosphoethanolamine, PE -phosphatidylethanolamine, P-MME - phosphomonomethylethanolamine; P-DME - phosphodimethylethanolamine, P-Cho - phosphocholine, Cho - choline, PC - phosphatidylcholine, CK - choline kinase, PMT - phosphoethanolamine N-methyltransferase, SAM - S-adenosylmethionine. Blue dashed arrow depicts the pathway of the methylation-dependent PC synthesis in humans. PEMT - Phosphatidylethanolamine N-methyltransferase. Solid lines depict pathway steps analyzed by proteomics in this study. Created in BioRender (Ermolaeva, M. (2026) https://BioRender.com/8k33rq6). **(b)** Quantitative RT-PCR analysis of *pmt-1* mRNA levels in *pmt-1* RNAi-treated worms, shown relative to control animals treated with empty vector (EV) RNAi. **(c)** Representative images of transgenic nematodes expressing GFP-tagged mitochondria in the body wall muscle (*myo-3p::gfpmit*) exposed to empty vector control or *pmt-1* RNAi as described in Figure 4e. Scale bar is 10μm. Images are from an independent replicate of the experiment quantified in Figure 4e. **(d)** Representative images of transgenic nematodes expressing GFP under the *hsp-6* promoter (*hsp-6p::GFP*) exposed to empty vector control or *mip-1* or *pmt-1* RNAi as described in Figure 4f. Each experiment was repeated at least 3 times, and one representative result is shown. In **b**, unpaired t-test was used for the statistical assessment. ****-p<0.0001, all p values are two-tailed. Each experiment was repeated at least 3 times, and one representative result is shown in each case. Mean, SEM and exact p values can be found in the Source Data file.

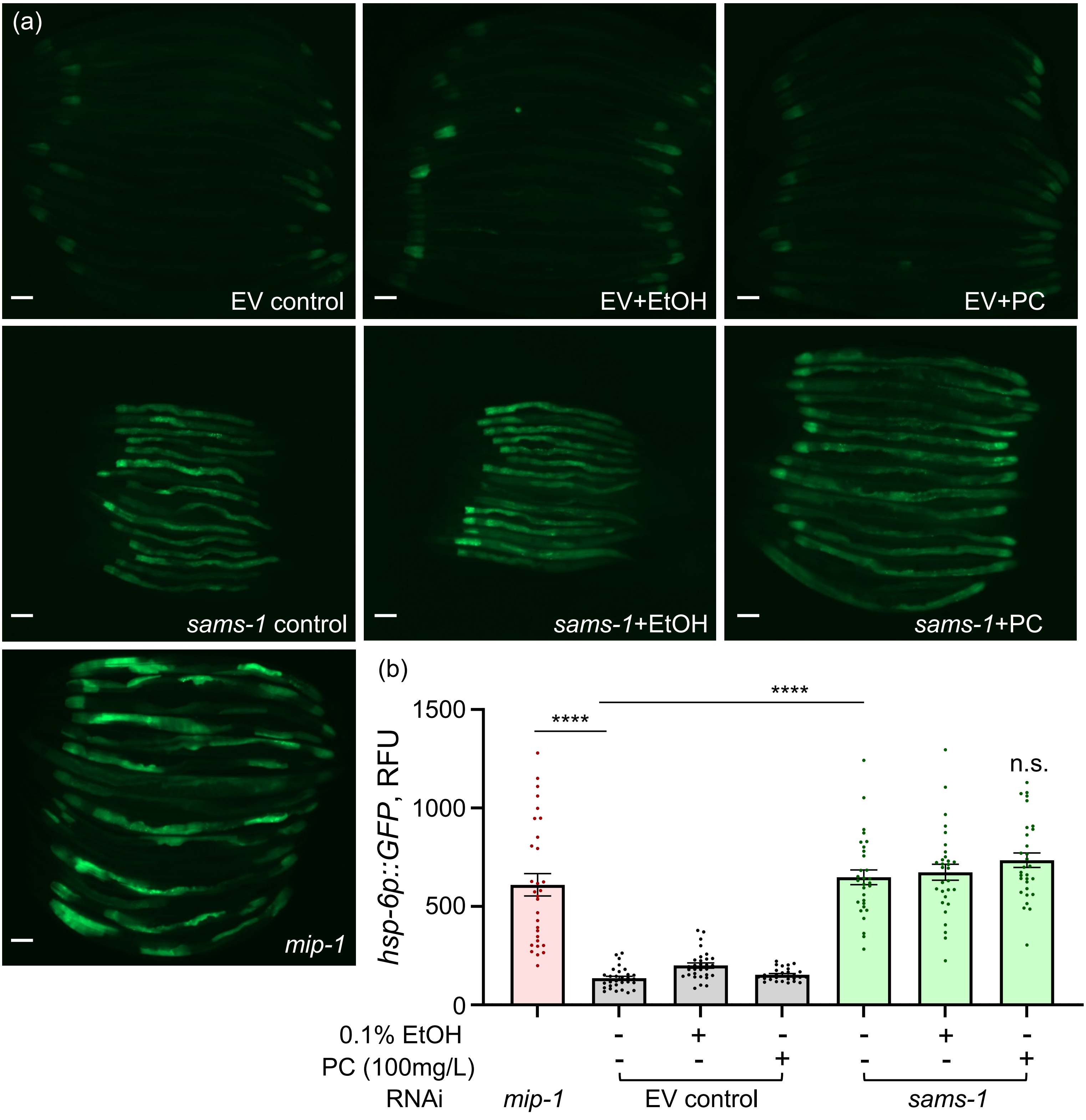

Supplementary Figure 4. Phosphatidylcholine supplementation does not suppress elevated UPR[mt] activity in *sams-1* knockdown animals. (a–b) Transgenic nematodes expressing GFP under control of the *hsp-6* promoter (*hsp-6p::GFP*) were age synchronized and exposed to control and *sams-1* RNAi from the L1 stage, 100mg/L phosphatidylcholine (PC) was provided from the L4 stage and 0.1% ethanol (EtOH) was used as a vehicle control for PC; *mip-1* RNAi was used as positive control for the UPR MT induction. GFP expression was assessed microscopically and quantified on AD2, n≥29 in each condition with precise n numbers reported in the Source Data file. Representative images are shown in **a** (scale bar is 100μm) and quantification in **b** with each dot corresponding to one animal. Statistics were assessed by using unpaired t-test with Welch's correction and two-tailed *p*-values were computed. The experiment was repeated 3 times, and one representative result is shown. n.s., not significant; ****-p<0.0001. Exact *p* values, mean and SEM values are presented in the Source Data file.

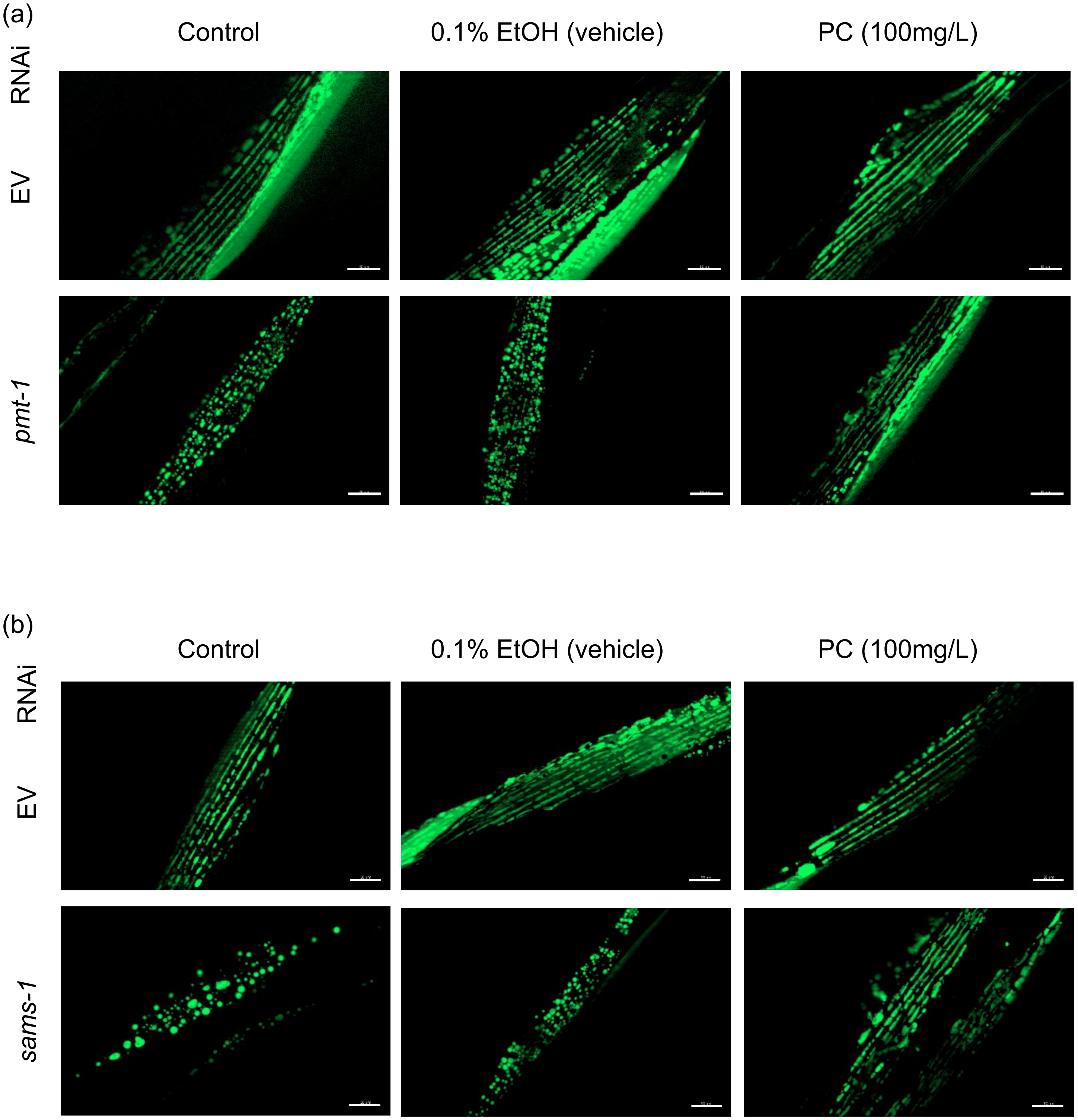

Supplementary Figure 5. Phosphatidylcholine supplementation rescues mitochondrial morphology defects of *sams-1* and *pmt-1* KD animals. (a-b) Representative images of age-synchronized *myo-3p::gfpmit* nematodes expressing GFP-tagged mitochondria in the body wall muscle and exposed to *pmt-1* RNAi **(a)** or *sams-1* RNAi **(b)** with and without phosphatidylcholine (PC, 100mg/L) supplementation and with 0.1% ethanol (EtOH) used as a vehicle control as in Figure 5a-b. Scale bar is 10μm. Images are from independent replicates of the experiments quantified in Figure 5a-b. Each experiment was repeated at least 3 times, and one representative result is shown in each case.

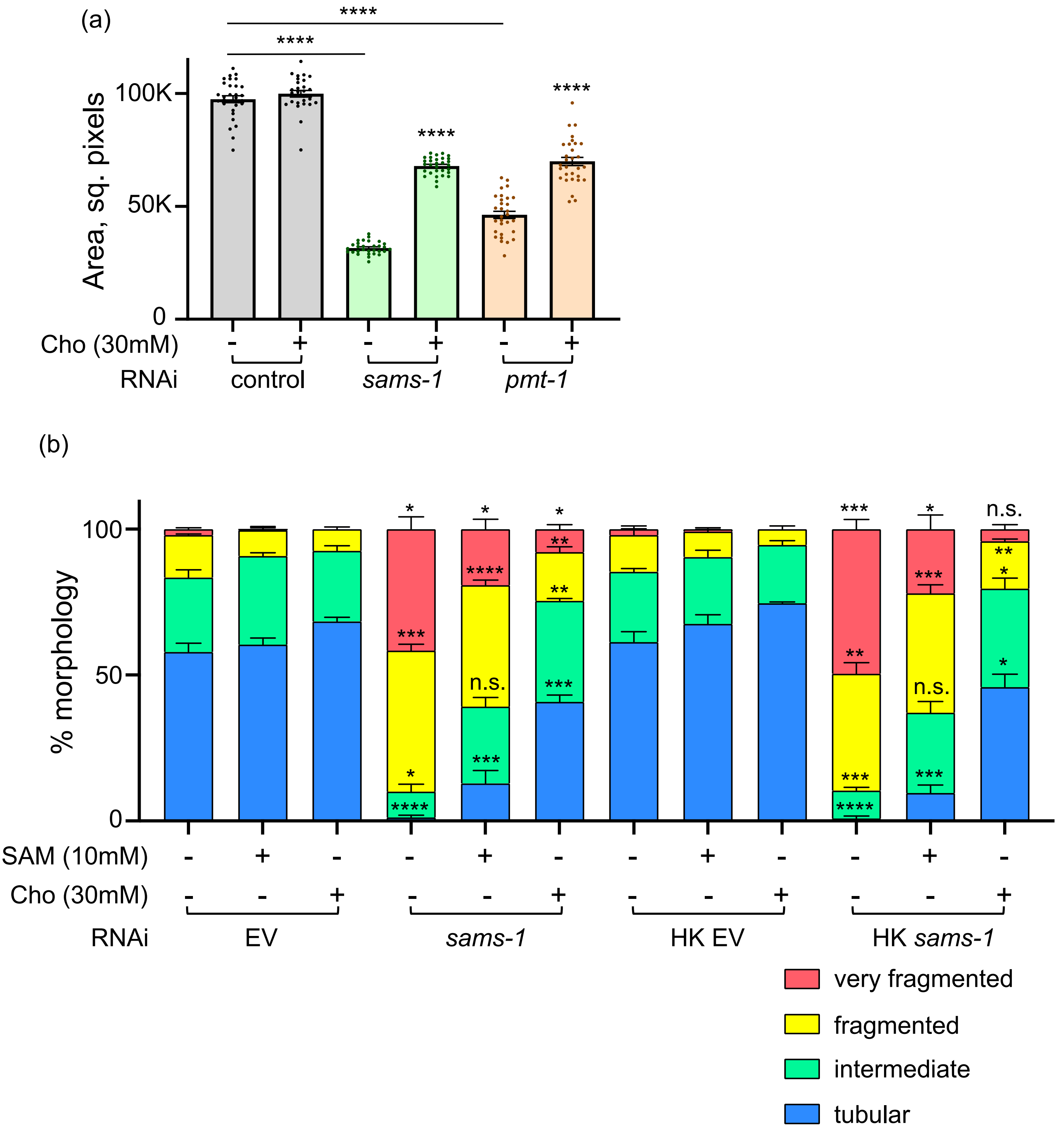

Supplementary Figure 6. Mitochondrial effects of SAM and choline supplementation are independent of microbial metabolism. (a) Age-synchronized *hsp-6p::GFP* nematodes were exposed to empty vector or *sams-1* or *pmt-1* RNAi from L1 stage and to 30mM choline (Cho) from L4 stage; body size (area) was measured on AD2, n≥28 in each condition with exact n numbers reported in the Source Data file; each dot corresponds to one animal. **(b)** Transgenic nematodes expressing GFP-tagged mitochondria in the body wall muscle *(myo-3p::gfpmit)* were exposed to live empty vector (EV) or *sams-1* RNAi from the L4 stage, and until gravid adulthood, and age-synchronized L1 progeny of these animals were collected by filtering. These L1 animals were then seeded and grown on either heat killed (HK) or live RNAi with or without 10mM SAM or 30mM choline (Cho) supplementation initiated at the L4 stage. Mitochondrial morphologies were scored on AD2, n=60 in all experimental groups. Experiments in **(a-b)** were repeated at least 3 times, and one representative result is shown in each case, unpaired t-test with Welch's correction was used for the statistical assessment. n.s., not significant; *-p<0.05; **-p<0.01; ***-p<0.001; ****-p<0.0001, all p values are two-tailed. Exact p values, mean and SEM values can be found in the Source Data file.

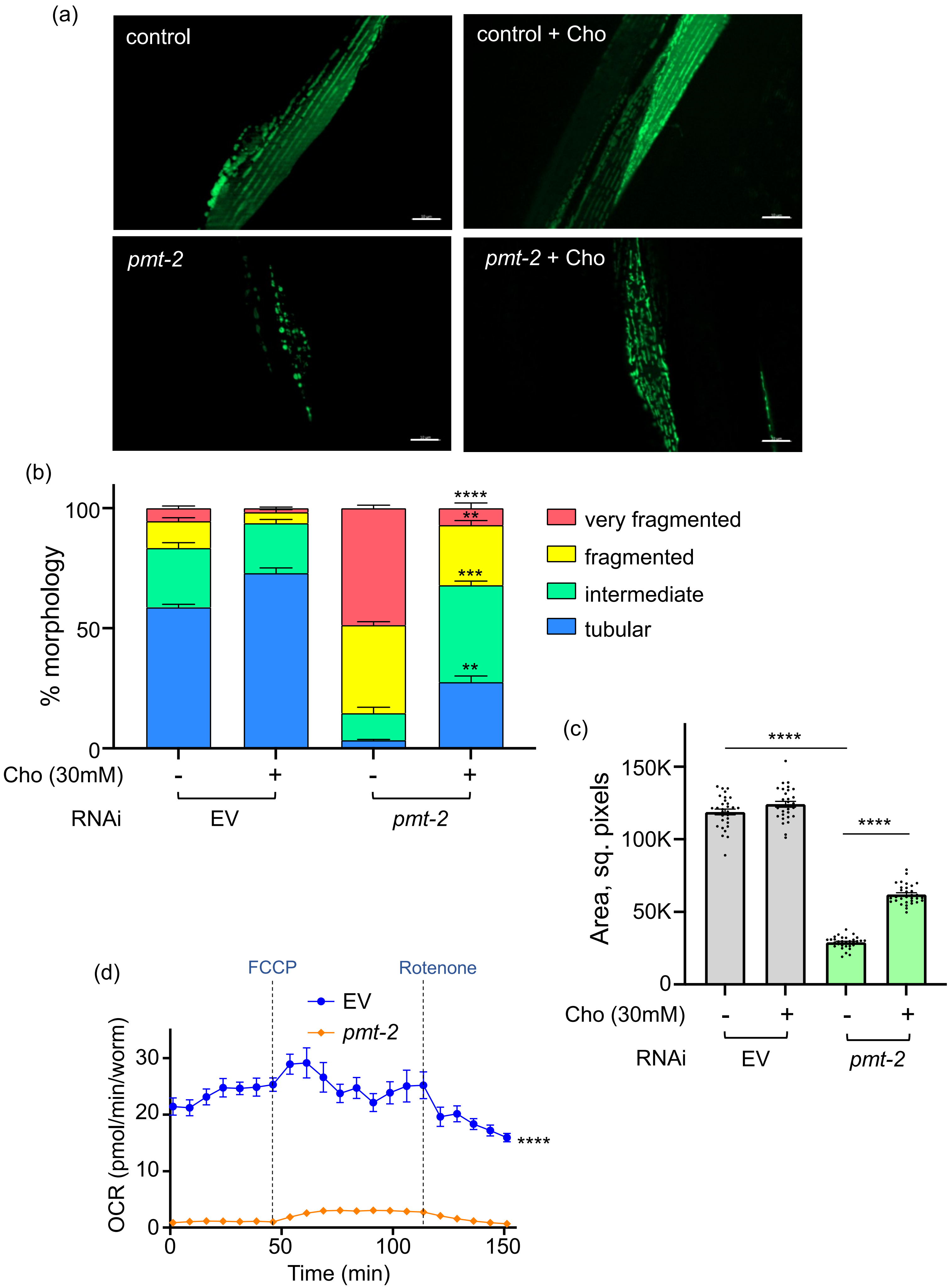

(a)
control
control + Cho
pmt-2
pmt-2 + Cho
(b)
% morphology
100
50
0
****
**
***
**
very fragmented
fragmented
intermediate
tubular
Cho (30mM)
-
+
-
+
RNAi
EV
pmt-2
(c)
Area, sq. pixels
150K
100K
50K
0
****
****
Cho (30mM)
-
+
-
+
RNAi
EV
pmt-2
(d)
FCCP
Rotenone
EV
pmt-2
OCR (pmol/min/worm)
30
20
10
0
****
0
50
100
150
Time (min)

Supplementary Figure 7. *pmt-2* deficiency phenocopies mitochondrial defects of *sams-1* and *pmt-1* KDs. (a–b) Transgenic nematodes expressing GFP-tagged mitochondria in the body wall muscle *(myo-3p::gfpmit)* were age-synchronized and exposed to *pmt-2* RNAi or empty vector control from L1 stage, 30mM choline (Cho) was supplemented from the L4 stage. Mitochondrial morphology was scored on AD2. Representative images are shown in **(a)**. Scale bar is 10μm. **(b)** Quantification as described in Figure 3b, n=60 in each condition. **(c)** Age-synchronized WT nematodes were exposed to RNAi and drug treatment as in **(a–b)** and body size (area) was measured on AD2, n≥30 in each condition with exact n numbers reported in the Source Data file; each dot corresponds to one animal. **(d)** Wild-type nematodes were exposed to EV or *pmt-2* RNAi from L1 stage and mitochondrial OCR was measured on AD2. n≥200 nematodes were used per condition with exact n numbers reported in the Source Data file. Data was normalized to number of worms. Each experiment in **(a-d)** was repeated at least 3 times, and one representative result is shown in each case. Statistics in **b–c** were assessed using unpaired t-test with Welch's correction. In **d**, area under the curve values were used to perform statistical analyses by unpaired t-test. n.s., not significant; **-*p*<0.01; ***-*p*<0.001; ****-*p*<0.0001, all *p* values are two-tailed. Exact *p* values, mean and SEM values can be found in the Source Data file.

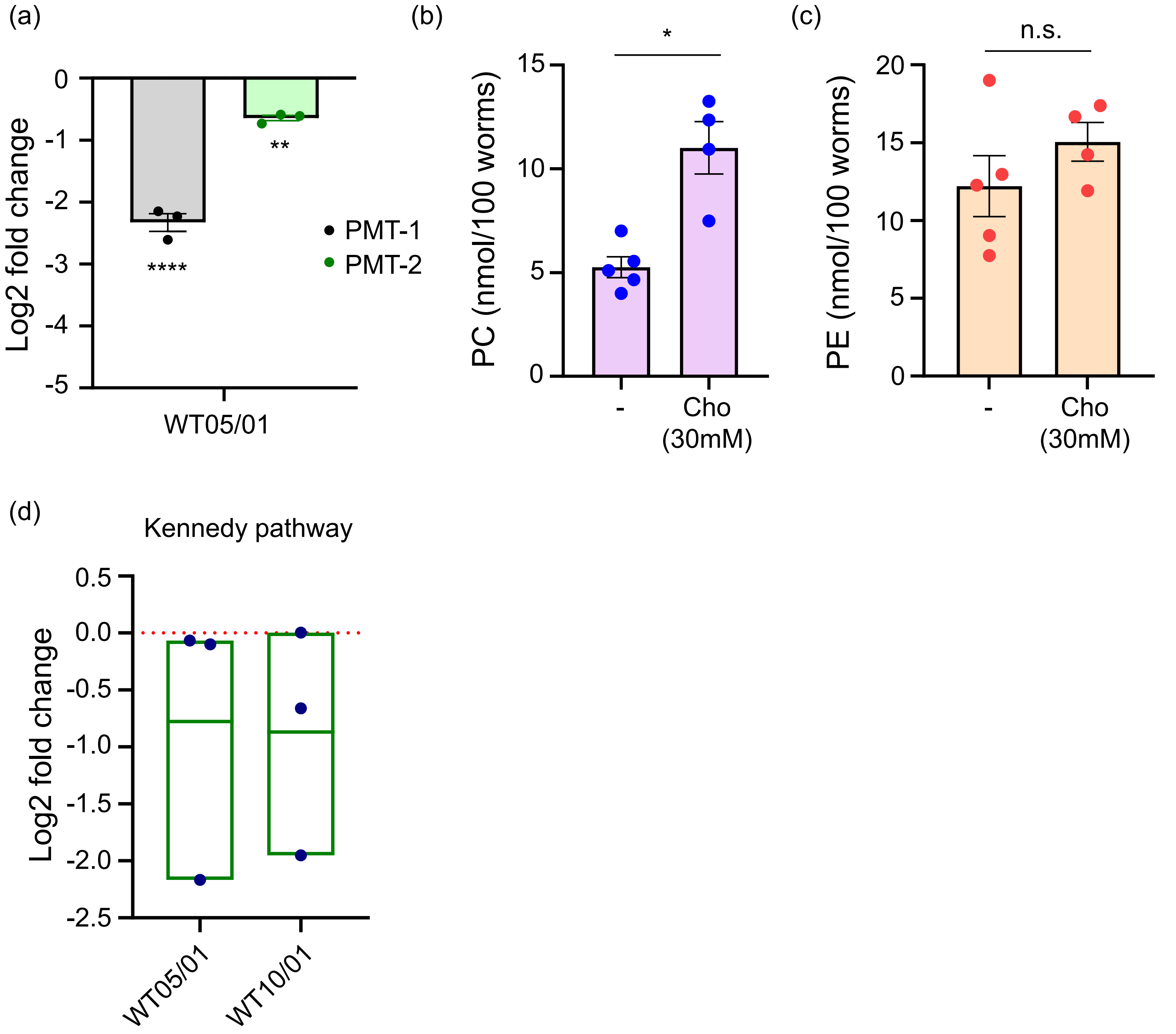

Supplementary Figure 8. Methylation-dependent phosphatidylcholine synthesis declines with age. (a) Proteomics samples were collected as described in Figure 1a and relative levels (expressed as Log2 fold change) of PMT-1 and PMT-2 proteins in post-reproductive (AD5 vs AD1) WT animals are shown. All relevant calculations can be found in Supplementary Data 4; each dot corresponds to one independent sample of 500 worms. **(b–c)** WT animals were age-synchronized and cultured until AD6 with or without choline (Cho) supplementation from the L4 stage, absolute intensities of phosphatidylcholine (PC) **(b)** or phosphatidylethanolamine (PE) **(c)** were measured as described in Figure 6d; values are normalized to the internal standard and the number of worms, and raw data can be found in Supplementary Data 28. Mean and SEM values are shown, each dot corresponds to one independent sample of 800 worms. **(d)** Proteomics samples were collected as described in Figure 1a and relative expression (Log2 fold change) of Kennedy pathway was measured in AD5 versus AD1 and AD10 versus AD1 WT animals. Each dot represents one protein and mean values are shown; raw data is shown in Supplementary Data 29. *p* values in **(a-d)** were computed by unpaired t-test with Welch's correction, 4-5 independent biological replicas were measured in **b**, **c** and 3 independent replicas were measured in **a**, **d**. n.s., not significant; *-*p*<0.05; **-*p*<0.01; ****-*p*<0.0001, all *p* values are two-tailed. Exact *p* values, n numbers, mean and SEM values can be found in the Source Data file.

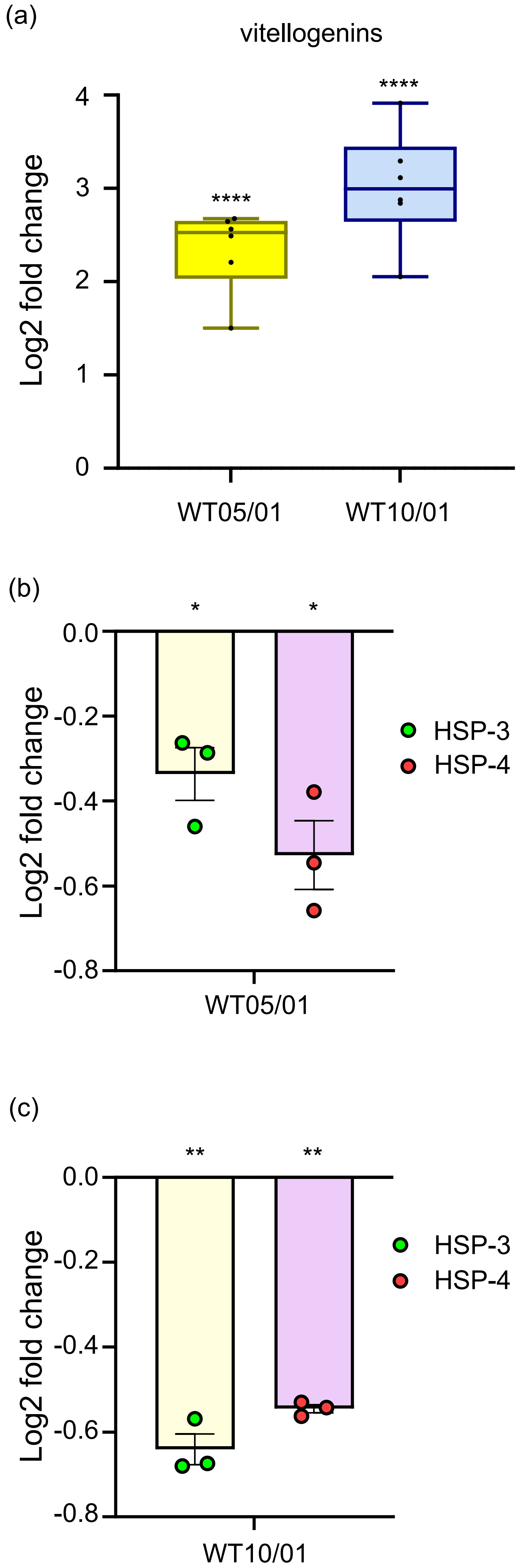

(a)
vitellogenins
****
****
Log2 fold change
4
3
2
1
0
WT05/01
WT10/01
(b)
*
*
0.0
-0.2
-0.4
-0.6
-0.8
Log2 fold change
HSP-3
HSP-4
WT05/01
(c)
**
**
0.0
-0.2
-0.4
-0.6
-0.8
Log2 fold change
HSP-3
HSP-4
WT10/01

Supplementary Figure 9. Elevated lipid droplet biomarkers do not correlate with UPR^{ER} activation in aged nematodes. Proteomics samples were collected as described in Figure 1a. **(a)** Box plots depicting relative expression (Log2 fold change) of vitellogenins in AD5 and AD10 versus AD1 WT nematodes are shown. Each dot represents one protein, median expression values are shown inside each box and whiskers highlight minimal and maximal values within each tested group. Statistics were assessed by one sample t-test and all relevant expression values can be found in Supplementary Data 30. Relative expression (Log2 fold change) of HSP-3 and HSP-4 ER chaperones is shown between AD5 and AD1 **(b)** and AD10 and AD1 **(c)** WT animals. Each dot represents one replica sample of 500 worms, mean and SEM values are presented and unpaired t-test was used to compute *p*-values. All relevant expression values and calculations can be found in Supplementary Data 4, 3 independent biological replicas were measured for all conditions. *-$p<0.05$; **-$p<0.01$; ****-$p<0.0001$, all *p* values are two-tailed and exact *p* values can be found in the Source Data file.

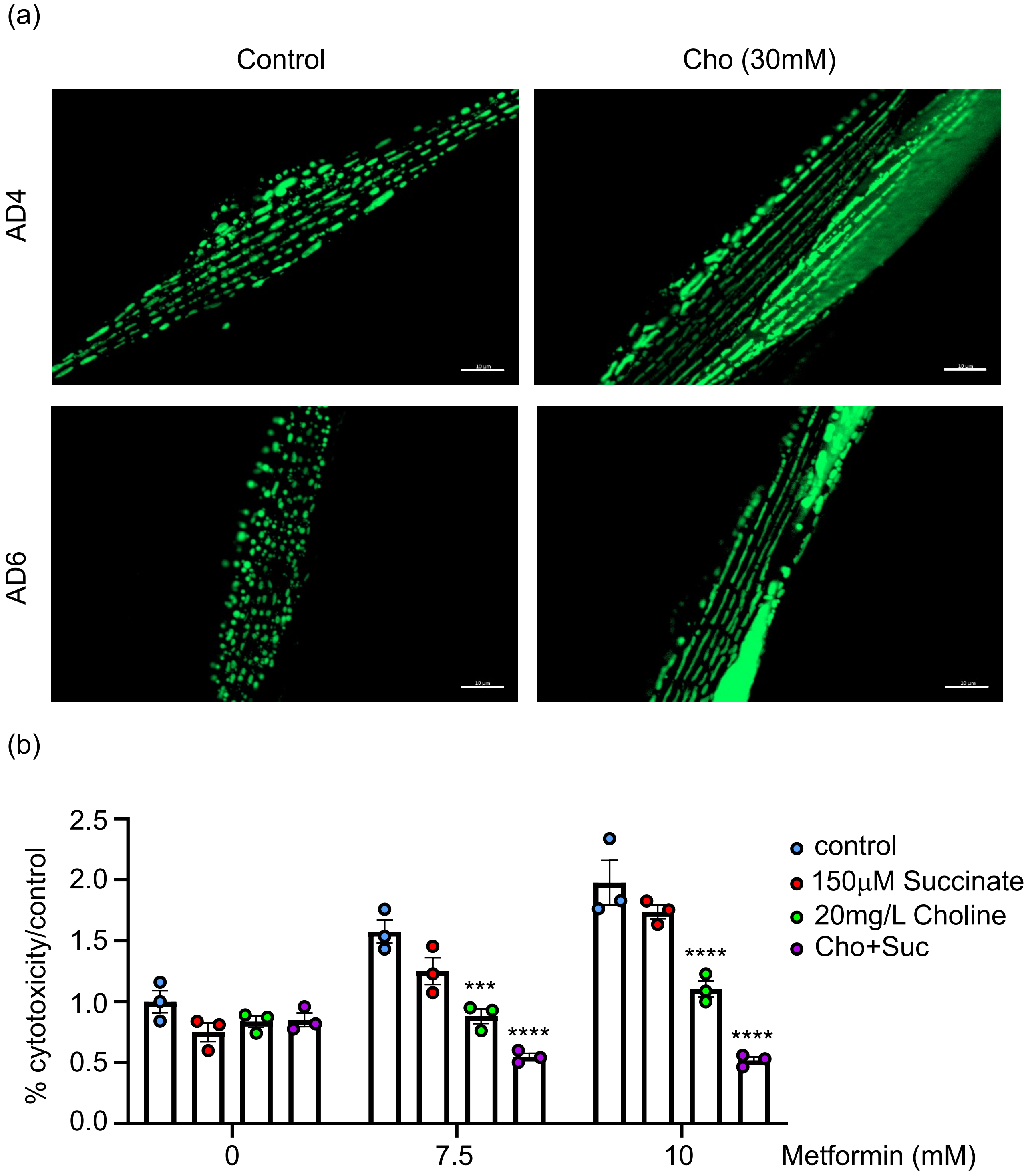

Supplementary Figure 10. Choline supplementation improves mitochondrial morphology and function in the context of aging and metabolic plasticity. (a) Age-synchronized nematodes were exposed to drug treatment for indicated times as in Figure 6e and representative images of mitochondria in body wall muscle cells are shown. Scale bar is 10μm. Images are from an independent replicate of the experiment quantified in Figure 6e. **(b)** The cytotoxicity (LDH assay) in BJ human skin fibroblasts was measured after metformin treatment with or without 150μM succinate, 20mg/L choline or a mix of choline and succinate for 24h as described in Figure 6g. n=3 in all cases, significance was assessed by multiple comparison t-test, mean and SEM values are presented; two-tailed *p* values were computed. *-$p<0.05$; **-$p<0.01$; ***-$p<0.001$. Each experiment was repeated at least 3 times, and one representative result is shown in each case. Exact *p* values can be found in the Source Data file.

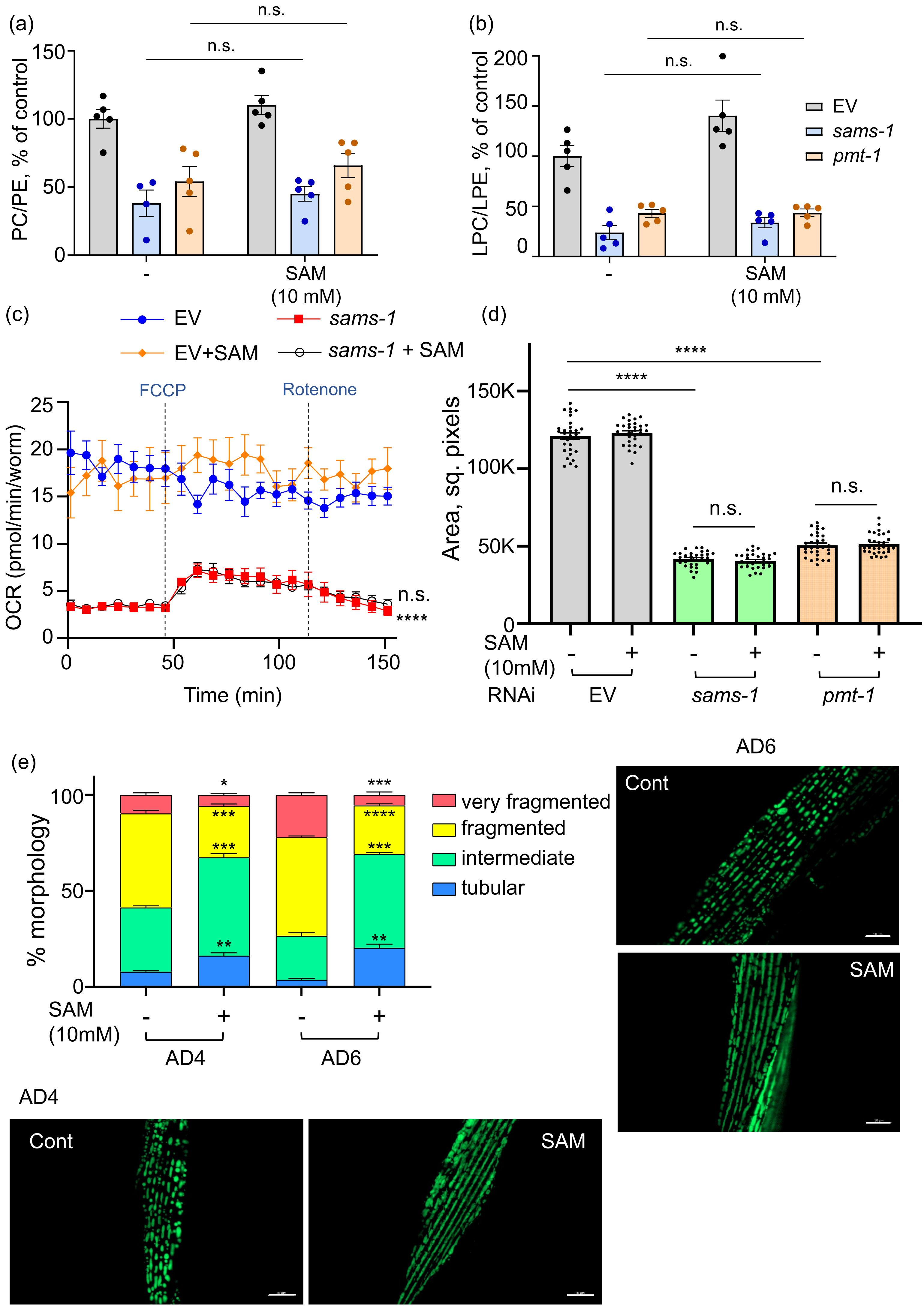
(a)
PC/PE, % of control
n.s.
SAM (10 mM)
(b)
LPC/LPE, % of control
EV
sams-1
pmt-1
(c)
EV+SAM
sams-1 + SAM
FCCP
Rotenone
OCR (pmol/min/worm)
Time (min)
n.s.
****
(d)
Area, sq. pixels
SAM (10mM)
RNAi
EV
sams-1
pmt-1
(e)
% morphology
very fragmented
fragmented
intermediate
tubular
AD4
AD6
Cont
SAM

Supplementary Figure 11. The effect of SAM supplementation on mitochondria is variable and likely hindered by chemical instability of SAM. Ratios of **(a)** phosphatidylcholine to phosphatidylethanolamine (PC/PE) and **(b)** lysophosphatidylcholine to lysophosphatidylethanolamine (LPC/LPE) in RNAi-treated animals. The same untreated empty vector (EV), *sams-1*, and *pmt-1* RNAi conditions as in Figure 6a–b are displayed here alongside the corresponding 10mM SAM-treated samples, which were measured in parallel. Data are normalized to the untreated, EV RNAi control. Mean and SEM values are shown, and raw data and calculations can be found in Supplementary Data 28; each dot corresponds to an independent sample of 800 worms. **(c)** Wild-type nematodes were exposed to EV or *sams-1* RNAi from the L1 stage and exposed to 10mM SAM from the L4 stage and until AD2. Mitochondrial OCR was measured on AD2, n≥150 nematodes with precise n number for each condition provided in the Source Data file. Data was normalized to the number of worms measured. **(d)** Age-synchronized wild type nematodes were exposed to RNAi as in Figure 5**a–b** and 10mM SAM was given from the L4 stage, body size (area) was measured on AD2, n≥28 with precise n number for each condition provided in the Source Data file; each dot corresponds to one animal. **(e)** Transgenic nematodes expressing GFP-tagged mitochondria in the body wall muscle (*myo-3p::gfpmit*) were age-synchronized and grown to indicated age with or without 10mM SAM supplementation from the L4 stage. Mitochondrial morphologies were scored on AD4 and AD6 as described in Figure 3b, n=60 in each condition. Representative images are shown. Scale bar is 10µm. In **c**, area under the curve values were used to perform statistical analyses by unpaired t-test. Significance in **a**, was measured by mixed-effects model with Tukey's post hoc test; in **b** - by two-way ANOVA with Tukey's post hoc test, in **d** and **e** - by unpaired t-test with Welch's correction. Two-tailed p values were computed. n.s., not significant; *-p<0.05; **-p<0.01; ***-p<0.001; ****-p<0.0001. Each experiment shown in **c–e** was repeated at least 3 times, and one representative result is presented in each case; in **a–b** 4–5 independent samples were measured. Exact p values, mean and SEM values can be found in the Source Data file.

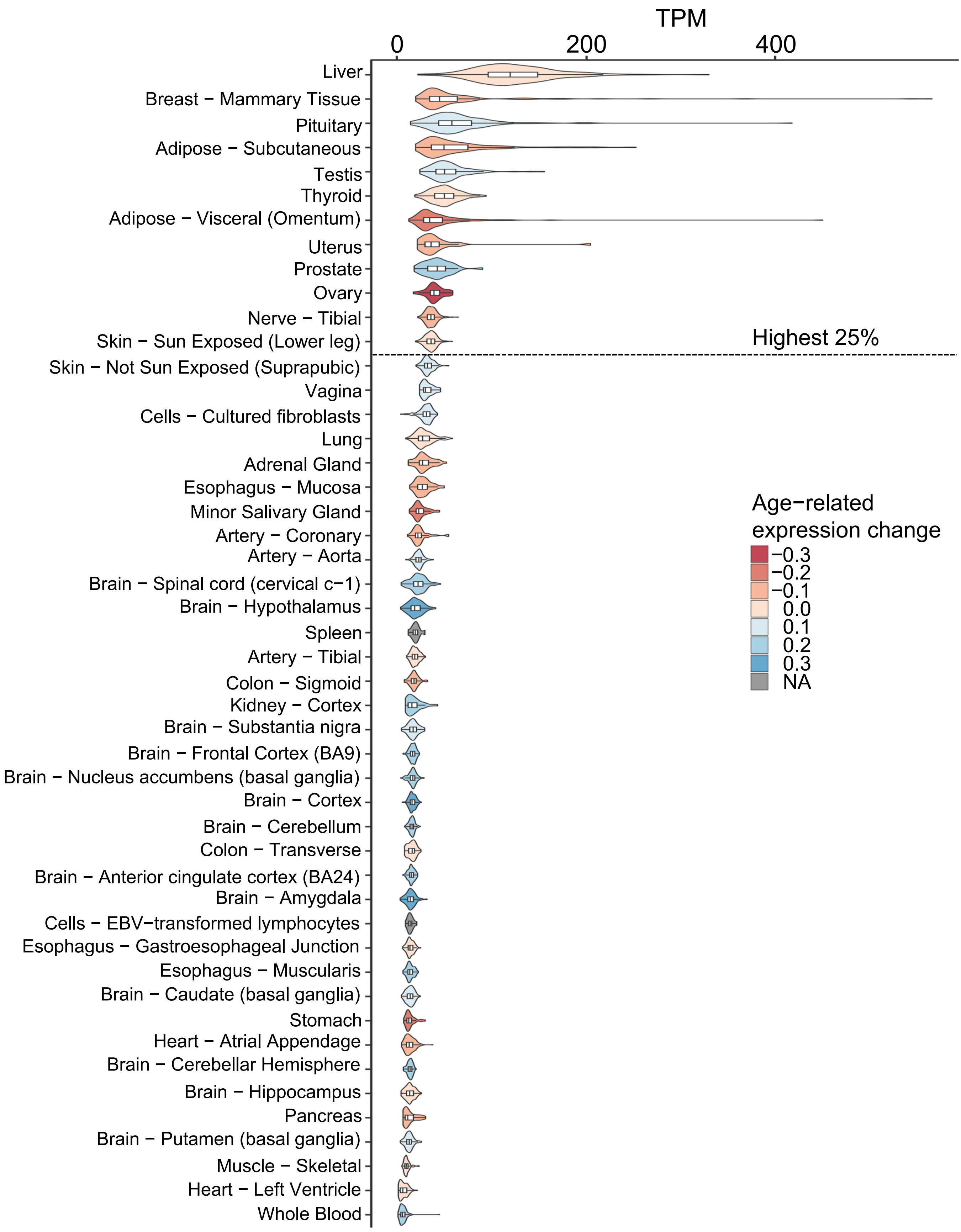

Supplementary Figure 12. Expression of PEMT across human tissues and age groups. The expression of human PEMT gene across tissues at different age was tested using the GTEx dataset (v8) as described in Figure 7b. Relative PEMT expression in different organs is depicted as transcript per million (TPM), and highest expressing tissues are outlined as top 25%. Expression changes associated with aging are color coded. The analysis was performed as described in the methods section, including code. All relevant values (expression and number of samples) can be found in Supplementary Data 31. NA denotes tissues with insufficient sample size to compute the correlation coefficient.

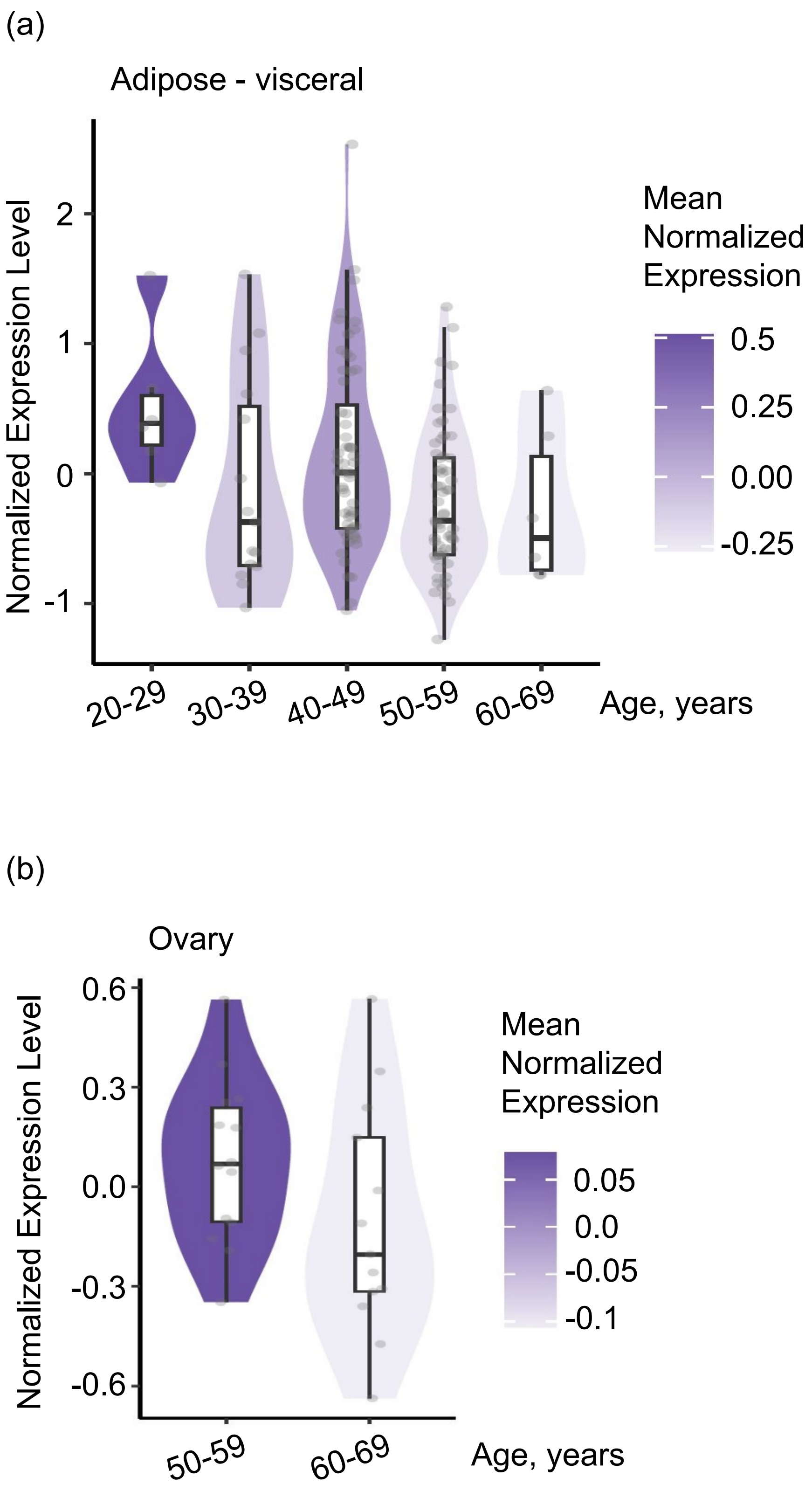

Supplementary Figure 13. Expression of PEMT declines during human aging in selected tissues. (a-b) The expression of human PEMT gene in the visceral adipose tissue **(a)** and ovary **(b)** at different age was tested using the GTEx dataset (v8) as described in Figure 7a. Normalized expression levels represent individual log2 transformed quantile normalized Transcripts Per Million (TPM) values corrected for sex and death circumstances using a linear model; median normalized PEMT expression in each age group is color coded and complete data can be found in Supplementary Data 34 and 35. Each dot corresponds to an independent sample and median values are shown. All relevant values (expression and number of samples) can be found in Supplementary Data 34 and 35.

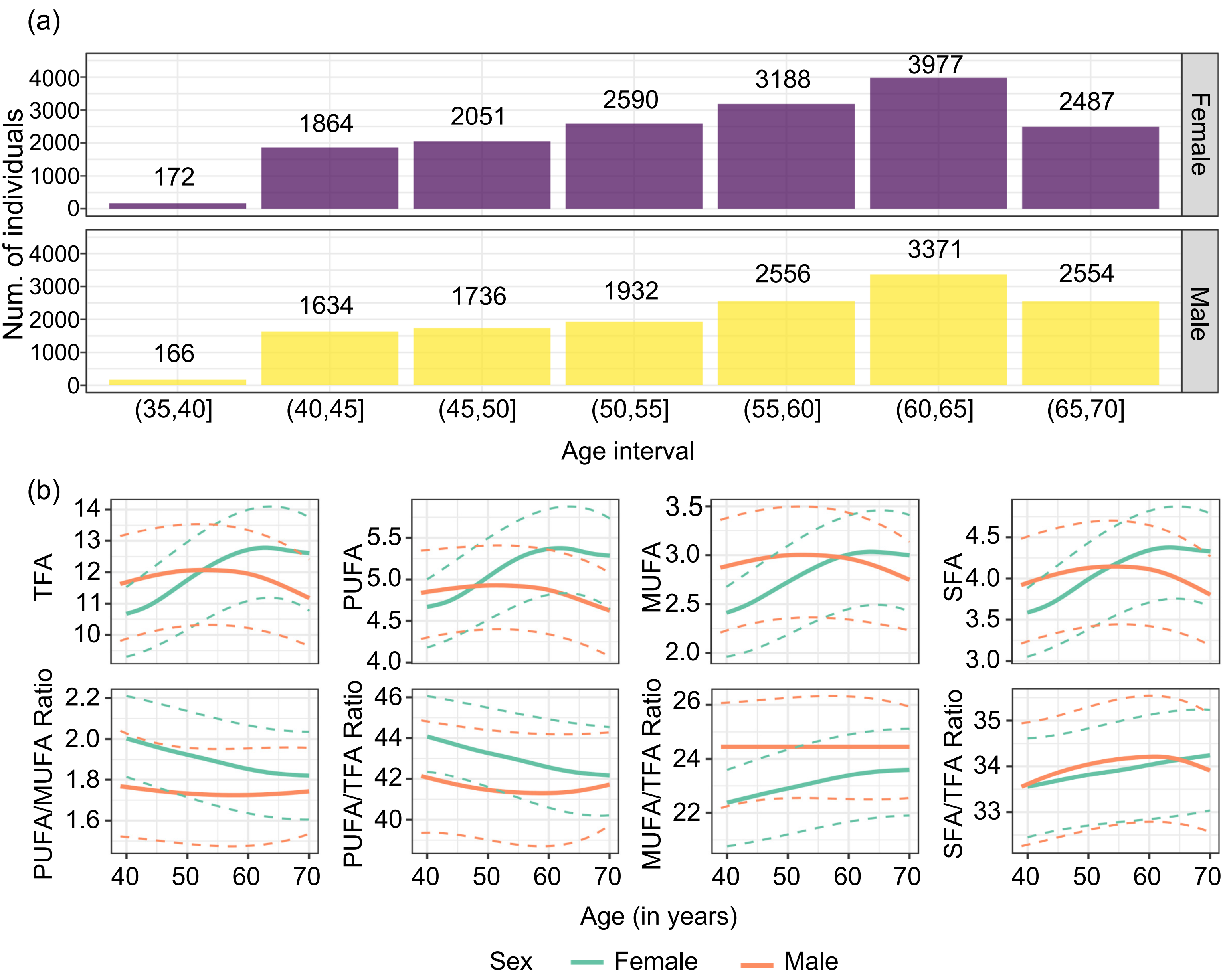

Supplementary Figure 14. The levels of lipids implicated in membrane fluidity are altered during human aging. **(a)** Barplots showing the number of UK Biobank samples analyzed in this study (the y-axis) across different age intervals (the x-axis) for females (top panel) and males (bottom panel) separately. The numbers on the bars show the exact number of samples with selection criteria described in the methods section. **(b)** Age-associated changes in EDTA plasma levels of indicated lipids measured in mmol/l. TFA – total fatty acids, PUFA – polyunsaturated fatty acids, MUFA – monounsaturated fatty acids, SFA – saturated fatty acids. The color coding reflects different sexes. The dashed lines show the first and fourth quartiles. The curves are created using the loess smoothing function. The values corresponding to panels **(a–b)** can be found in Supplementary Data 36 and 38 respectively.

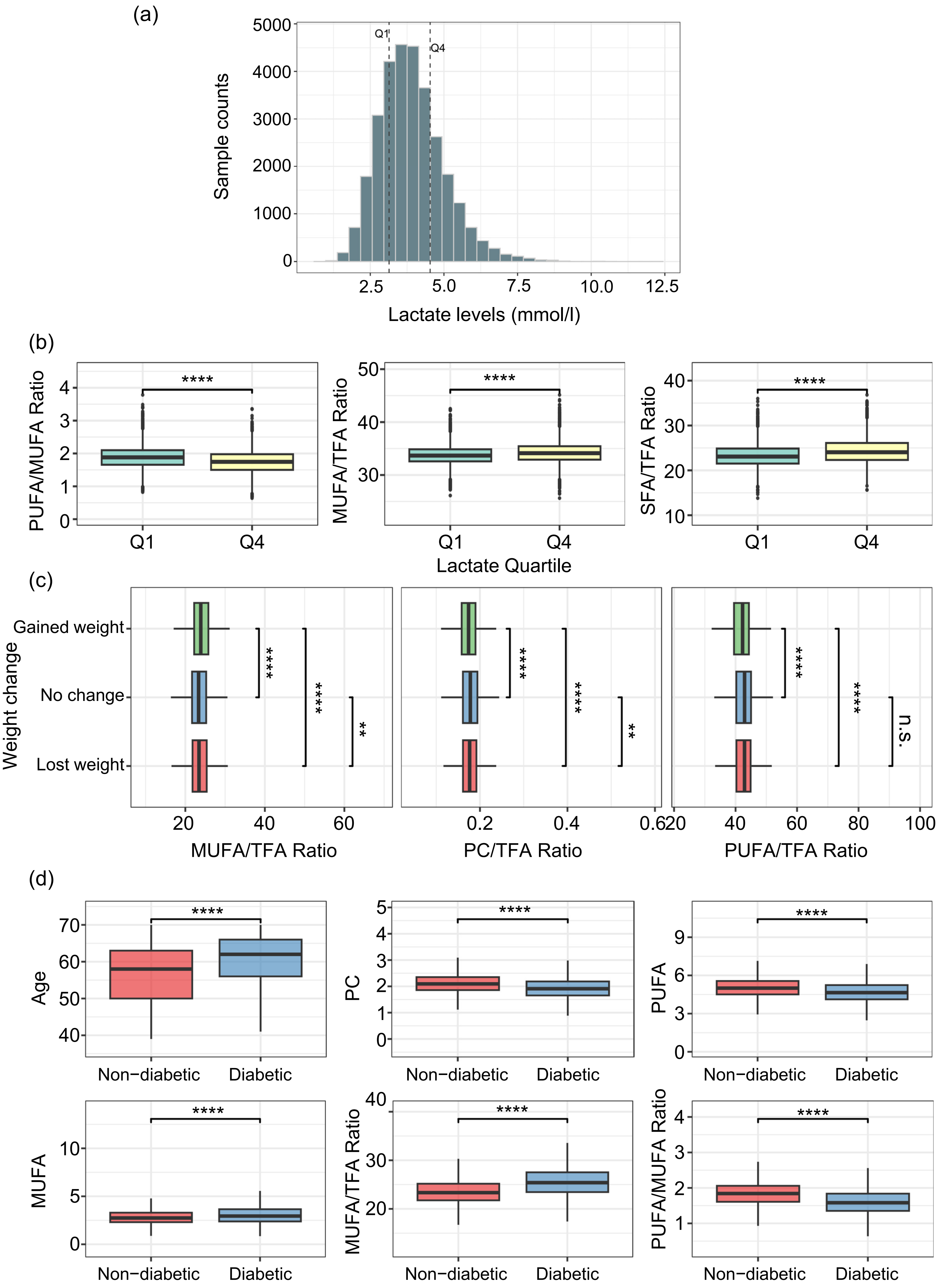

(a)
Sample counts
Lactate levels (mmol/l)
Q1
Q4
5000
4000
3000
2000
1000
0
2.5
5.0
7.5
10.0
12.5
(b)
PUFA/MUFA Ratio
MUFA/TFA Ratio
SFA/TFA Ratio
Lactate Quartile
Q1
Q4
****
(c)
Weight change
Gained weight
No change
Lost weight
MUFA/TFA Ratio
PC/TFA Ratio
PUFA/TFA Ratio
****
**
n.s.
(d)
Age
PC
PUFA
MUFA
MUFA/TFA Ratio
PUFA/MUFA Ratio
Non−diabetic
Diabetic
****

Supplementary Figure 15. Higher PC and PUFA levels correlate with improved metabolic health in humans. (a) Histogram of lactate levels (mmol/l; n = 30,239). Vertical dashed lines indicate the 25th (Q1) and 75th (Q4) percentiles (3.14 and 4.50 mmol/l, respectively). **(b)** Samples were sorted based on serum lactate levels. Boxplots show the distribution of polyunsaturated fatty acids to monounsaturated fatty acids ratio (PUFA/MUFA Ratio, left panel), monounsaturated fatty acids to total fatty acids ratio (MUFA/TFA Ratio, middle panel), and saturated fatty acids to total fatty acids ratio (SFA/TFA Ratio, left panel) for samples in the top 25% (i.e., Q1) and bottom 25% (i.e., Q4) lactate quartiles. The groups were compared using the Wilcoxon rank-sum test. Background values are shown in Supplementary Data 40. **(c)** Boxplots displaying the ratios of monounsaturated fatty acids to total fatty acids (MUFA/TFA, left panel), phosphatidylcholine to total fatty acids (PC/TFA, middle panel), and polyunsaturated fatty acids to total fatty acids (PUFA/TFA, right panel) across individuals reporting weight gain, no weight change, or weight loss compared to one year ago. Background values are shown in Supplementary Data 42. **(d)** Boxplots show the distribution of individual ages (top left panel), phosphatidylcholine (PC) levels measured in mmol/l (top middle), polyunsaturated fatty acids (PUFA) levels in mmol/l (top right), monounsaturated fatty acids (MUFA) levels in mmol/l (bottom left), monounsaturated fatty acids to total fatty acids ratio (MUFA/TFA Ratio, bottom middle), and polyunsaturated fatty acids to monounsaturated fatty acids ratio (PUFA/MUFA Ratio, bottom right panel) for diabetic (n=3,100) and non-diabetic (n=27,169) individuals. Background values are shown in Supplementary data 43. The groups were compared using the Wilcoxon rank-sum test. The asterisk throughout the figure shows significance: n.s., not significant; *-p<0.05; **-p<0.01; ***-p<0.001 and ****-p<0.0001; all p values are two-tailed. Exact p values, mean and SEM values can be found in the Source Data file.

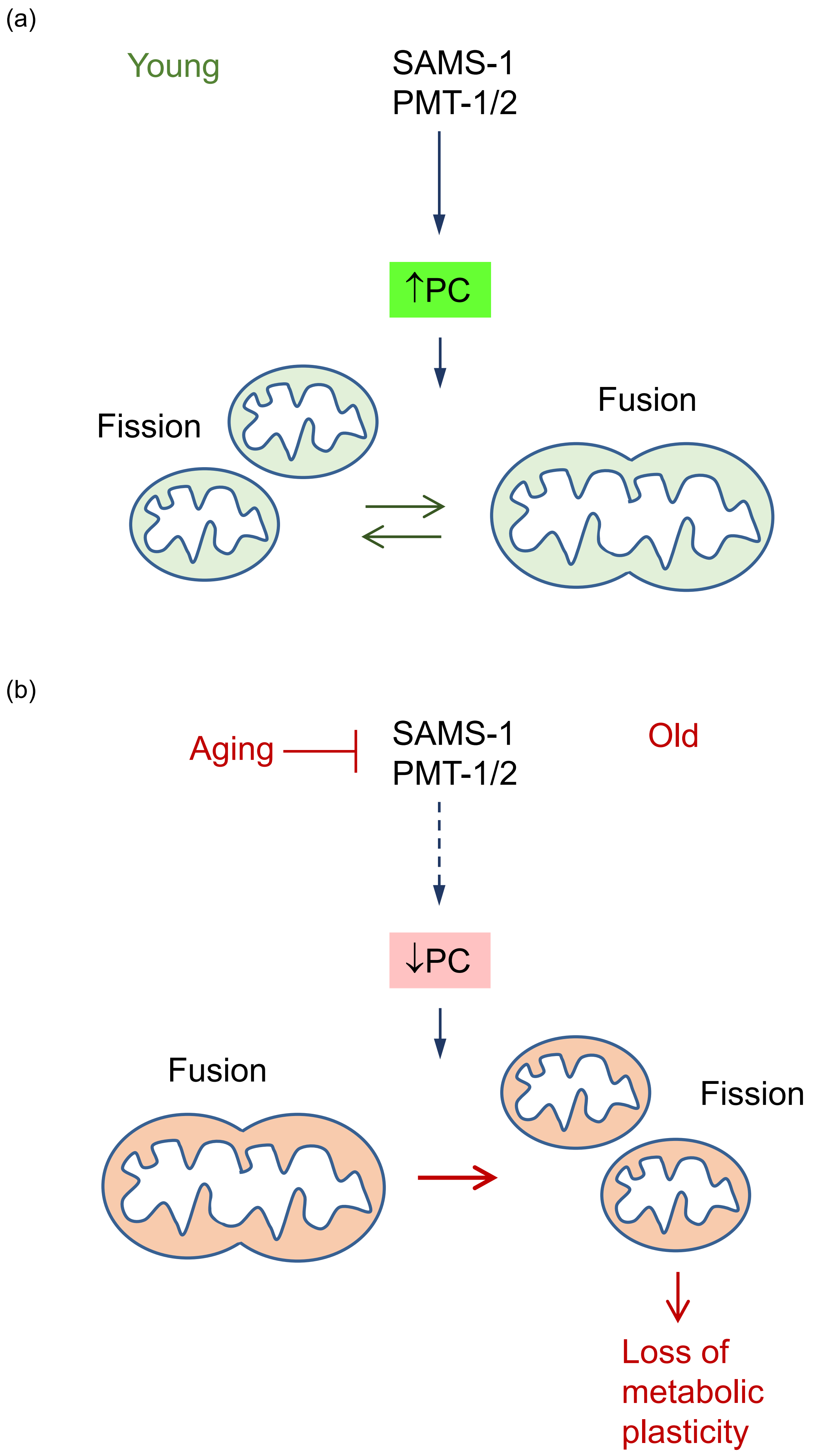
(a)
Young
SAMS-1
PMT-1/2
↑PC
Fission
Fusion
(b)
Aging
SAMS-1
PMT-1/2
Old
↓PC
Fusion
Fission
Loss of
metabolic
plasticity

Supplementary Figure 16. Aging-triggered lipidome remodeling interferes with mitochondrial fusion and metabolic plasticity. Our data demonstrate that the aging-related decline in SAM-dependent phosphatidylcholine (PC) synthesis impairs mitochondrial fusion, thereby contributing to mitochondrial dysfunction and reduced metabolic plasticity in later life. Notably, these effects are likely driven by the essential roles of PC and its derivative lysophosphatidylcholine in maintaining membrane curvature and fluidity—key biophysical properties required for proper mitochondrial dynamics.
