# Supplementary material for: Aging-associated decline of phosphatidylcholine synthesis is a malleable trigger of natural mitochondrial aging
Source: Nat Commun. 2026 Apr 18;17:3589. doi: 10.1038/s41467-026-71508-7 (PMC13091796; doi:10.1038/s41467-026-71508-7)
Supplement: Supplementary file 2 — Description of Additional Supplementary Files [file 41467_2026_71508_MOESM2_ESM.pdf]

**Title:** Supplementary Data 1.

**Description:** Proteomic profiling of *C. elegans* wild-type (N2) and the mitochondrial mutants *isp-1(qm150)* and *clk-1(qm30)*. Proteomes were quantified on adulthood days 1, 5, and 10. Absolute abundance values for all identified protein groups are provided. n = 3 biological replicates per condition.

**Title:** Supplementary Data 2.

**Description:** Proteomic profiling of *C. elegans* wild-type (N2) and the mitochondrial mutants *isp-1(qm150)* and *clk-1(qm30)*. Relative comparisons between experimental conditions are shown across the entire dataset, including all relevant statistics and parameters. Calculations are based on the absolute abundance values reported in Supplementary Data 1.

**Title:** Supplementary Data 3.

**Description:** Relative abundance of ribosomal proteins in *C. elegans clk-1(qm30)* mutants compared with wild-type (N2) animals on adulthood day 1. Differences in protein abundance were assessed using a rank-sum test.

**Title:** Supplementary Data 4.

**Description:** Fold changes (FCs) of the indicated proteins between specified conditions, calculated using absolute abundance values from Supplementary Data 1. The Excel sheet includes the formulas used for each calculation.

**Title:** Supplementary Data 5.

**Description:** Relative changes in protein abundance between adulthood day 5 and adulthood day 1 in wild-type (N2) animals. Only statistically significant changes are shown, filtered by an absolute average $log_2$ fold change ratio $\geq 0.58$ and a Q-value $< 0.05$. This dataset represents a subset of Supplementary Data 2 and was extracted for the analyses presented in Supplementary Data 7-11.

**Title:** Supplementary Data 6.

**Description:** Relative changes in protein abundance between adulthood day 10 and adulthood day 1 in wild-type (N2) animals. Only statistically significant changes are shown, filtered by an

absolute average $\log_2$ fold change ratio $\geq 0.58$ and a Q-value $< 0.05$. This dataset represents a subset of Supplementary Data 2 and was extracted for the analyses presented in Supplementary Data 7-11.

**Title:** Supplementary Data 7.

**Description:** Venn analysis of protein sets derived from Supplementary Data 5 and 6, using gene names as both input and output.

**Title:** Supplementary Data 8.

**Description:** WormBase identifiers corresponding to the Venn analysis results from Supplementary Data 7. Data are presented in three columns, with column headers reflecting the respective Venn outputs. These identifiers are provided to enable independent WormCat analysis.

**Title:** Supplementary Data 9.

**Description:** WormCat analysis of proteins differentially regulated during early wild-type aging (N2 D5 column in Supplementary Data 8). Results are summarized across all three WormCat category levels. Bonferroni correction was applied to account for multiple comparisons. The complete analysis, including native WormCat graphics, is provided in Supplementary Data 46.

**Title:** Supplementary Data 10.

**Description:** WormCat analysis of proteins differentially regulated during both early and late wild-type aging (N2 D10, N2 D5 column in Supplementary Data 8). Results are summarized across all three WormCat category levels. Bonferroni correction was applied to account for multiple comparisons. The complete analysis, including native WormCat graphics, is provided in Supplementary Data 46.

**Title:** Supplementary Data 11.

**Description:** WormCat analysis of proteins differentially regulated during late wild-type aging (N2 D10 column in Supplementary Data 8). Results are summarized across all three WormCat category levels. Bonferroni correction was applied to account for multiple comparisons. The complete analysis, including native WormCat graphics, is provided in Supplementary Data 46.

**Title:** Supplementary Data 12.

**Description:** Relative changes in protein abundance between adulthood day 10 and adulthood day 1 in *clk-1(qm30)* animals. Only statistically significant changes are shown, filtered by an absolute average $\log_2$ fold change ratio $\geq 0.58$ and a Q-value $< 0.05$. This dataset represents a subset of Supplementary Data 2 and was extracted for the analyses presented in Supplementary Data 13-16.

**Title:** Supplementary Data 13.

**Description:** Venn analysis of protein sets derived from Supplementary Data 6 and 12, using gene names as both input and output.

**Title:** Supplementary Data 14.

**Description:** WormBase identifiers corresponding to the Venn analysis results from Supplementary Data 13. Data are presented in two columns, with column headers reflecting the respective Venn outputs. These identifiers are provided to enable independent WormCat analysis.

**Title:** Supplementary Data 15.

**Description:** WormCat analysis of proteins commonly regulated between wild-type and *clk-1(qm30)* animals in late age (N2 D10/D1, clk-1 D10/D1 column in Supplementary Data 14). Results are summarized across all three WormCat category levels. Bonferroni correction was applied to account for multiple comparisons. The complete analysis, including native WormCat graphics, is provided in Supplementary Data 46. The percentage of metabolic RGSs in category 3 data (metabolic contribution) is calculated at the bottom of the sheet.

**Title:** Supplementary Data 16.

**Description:** WormCat analysis of proteins differentially regulated during late *clk-1(qm30)* aging (*clk-1* D10/D1 column in Supplementary Data 14). Results are summarized across all three WormCat category levels. Bonferroni correction was applied to account for multiple comparisons. The complete analysis, including native WormCat graphics, is provided in Supplementary Data 46.

**Title:** Supplementary Data 17.

**Description:** Relative changes in protein abundance between adulthood day 10 and adulthood day 1 in *isp-1(qm150)* animals. Only statistically significant changes are shown, filtered by an absolute average $\log_2$ fold change ratio $\geq 0.58$ and a Q-value $< 0.05$. This dataset represents a subset of Supplementary Data 2 and was extracted for the analyses presented in Supplementary Data 18-21.

**Title:** Supplementary Data 18.

**Description:** Venn analysis of protein sets derived from Supplementary Data 6 and 17, using gene names as both input and output.

**Title:** Supplementary Data 19.

**Description:** WormBase identifiers corresponding to the Venn analysis results from Supplementary Data 18. Data are presented in two columns, with column headers reflecting the respective Venn outputs. These identifiers are provided to enable independent WormCat analysis.

**Title:** Supplementary Data 20.

**Description:** WormCat analysis of proteins commonly regulated between wild-type and *isp-1(qm150)* animals in late age (N2 D10/D1, isp-1 D10/D1 column in Supplementary Data 19). Results are summarized across all three WormCat category levels. Bonferroni correction was applied to account for multiple comparisons. The complete analysis, including native WormCat graphics, is provided in Supplementary Data 46. The percentage of metabolic RGSs in category 3 data (metabolic contribution) is calculated at the bottom of the sheet.

**Title:** Supplementary Data 21.

**Description:** WormCat analysis of proteins differentially regulated during late *isp-1(qm150)* aging (isp-1 D10/D1 column in Supplementary Data 19). Results are summarized across all three WormCat category levels. Bonferroni correction was applied to account for multiple comparisons. The complete analysis, including native WormCat graphics, is provided in Supplementary Data 46.

**Title:** Supplementary Data 22.

**Description:** Venn analysis of protein sets derived from Supplementary Data 12 and 17, using gene names as both input and output.

**Title:** Supplementary Data 23.

WormBase identifiers corresponding to the Venn analysis results from Supplementary Data 22. Data are presented in three columns, with column headers reflecting the respective Venn outputs. These identifiers are provided to enable independent WormCat analysis.

**Title:** Supplementary Data 24.

**Description:** WormCat analysis of proteins commonly regulated between *clk-1(qm30)* and *isp-1(qm150)* animals in late age (clk-1 D10/D1, isp-1 D10/D1 column in Supplementary Data 23). Results are summarized across all three WormCat category levels. Bonferroni correction was applied to account for multiple comparisons. The complete analysis, including native WormCat graphics, is provided in Supplementary Data 46. The percentage of metabolic RGSs in category 3 data (metabolic contribution) is calculated at the bottom of the sheet.

**Title:** Supplementary Data 25.

**Description:** WormCat analysis of proteins differentially regulated during late *clk-1(qm30)* aging (clk-1 D10/D1 column in Supplementary Data 23). Results are summarized across all three WormCat category levels. Bonferroni correction was applied to account for multiple comparisons. The complete analysis, including native WormCat graphics, is provided in Supplementary Data 46.

**Title:** Supplementary Data 26.

**Description:** WormCat analysis of proteins differentially regulated during late *isp-1(qm150)* aging (isp-1 D10/D1 column in Supplementary Data 23). Results are summarized across all three WormCat category levels. Bonferroni correction was applied to account for multiple comparisons. The complete analysis, including native WormCat graphics, is provided in Supplementary Data 46.

**Title:** Supplementary Data 27.

**Description:** Relative changes in protein abundance between adulthood day 10 and adulthood day 1 in wild-type (N2) animals. The most significant changes are shown, filtered by an absolute average $\log_2$ fold change ratio ≥ 2.5 and sorted by ascending Q-value. This dataset represents a

subset of Supplementary Data 2. Downregulated proteins are marked in blue and upregulated proteins in red.

**Title:** Supplementary Data 28.

**Description:** The complete lipidomics dataset (absolute values in nmol per 100 worms) is presented, and all calculations relevant to the corresponding figures are included on the left side of the sheet. n = 5 for each condition.

**Title:** Supplementary Data 29.

**Description:** Relative changes in the abundance of Kennedy pathway proteins in wild-type *C. elegans* are shown for adulthood day 5 versus adulthood day 1 (upper rows) and adulthood day 10 versus adulthood day 1 (lower rows), including all relevant statistical analyses and parameters.

**Title:** Supplementary Data 30.

**Description:** Relative changes in the protein abundance of vitellogenins in wild-type *C. elegans* are shown for adulthood day 5 versus adulthood day 1 (upper rows) and adulthood day 10 versus adulthood day 1 (lower rows), including all relevant statistical analyses and parameters.

**Title:** Supplementary Data 31.

**Description:** The expression of human PEMT gene across tissues at different age was tested using the GTEx dataset (v8) as described in Figure 7b. Relative PEMT expression across organs is shown as transcripts per million (TPM). Individual sample values and the mean expression for each tissue are displayed. Tissues are grouped into the highest-expressing 25% and the lowest-expressing 75%.

**Title:** Supplementary Data 32.

**Description:** Tissues with high (top 25%) and low (bottom 75%) PEMT expression, as defined in Supplementary Data 31, were analyzed for age-related changes by computing Spearman's correlations between age and expression levels across all available time points. The resulting Spearman's rho values are shown for each tissue.

**Title:** Supplementary Data 33.

**Description:** Expression data of the human PEMT gene in subcutaneous adipose tissue across age is shown. PEMT expression was analyzed using the GTEx dataset (v8; n = 192 samples), as described in Figure 7a. Normalized expression values represent individual $log_2$-transformed, quantile-normalized transcripts per million (TPM), corrected for sex and circumstances of death using a linear model.

**Title:** Supplementary Data 34.

**Description:** Expression data of the human PEMT gene in visceral adipose tissue across age is shown. PEMT expression was analyzed using the GTEx dataset (v8; n = 192 samples), as described in Figure 7a. Normalized expression values represent individual $log_2$-transformed, quantile-normalized transcripts per million (TPM), corrected for sex and circumstances of death using a linear model.

**Title:** Supplementary Data 35.

**Description:** Expression data of the human PEMT gene in ovarian tissue across age is shown. PEMT expression was analyzed using the GTEx dataset (v8; n = 192 samples), as described in Figure 7a. Normalized expression values represent individual $log_2$-transformed, quantile-normalized transcripts per million (TPM), corrected for sex and circumstances of death using a linear model.

**Title:** Supplementary Data 36.

**Description:** Overview of UK Biobank samples analyzed in this study across age intervals. The respective numbers of male and female samples are shown.

**Title:** Supplementary Data 37.

**Description:** Overview of NMR metabolomics data from the UK Biobank cohort used in this study, focusing on phosphatidylcholine (PC) levels and PC-to-total fatty acid (TFA) ratios across sexes and age groups.

**Title:** Supplementary Data 38.

**Description:** Overview of NMR metabolomics data from the UK Biobank cohort analyzed in this study, showing levels of total fatty acids (TFA), polyunsaturated (PUFA), monounsaturated

(MUFA), and saturated fatty acids (SFA), as well as their respective ratios across sexes and age groups.

**Title:** Supplementary Data 39.

**Description:** Distribution of fatty acid ratios across serum lactate quartiles (NMR metabolomics, UK Biobank data), focusing on PC/TFA and PUFA/TFA ratios.

**Title:** Supplementary Data 40.

**Description:** Distribution of fatty acid ratios across serum lactate quartiles (NMR metabolomics, UK Biobank data), focusing on PUFA/MUFA, MUFA/TFA and SFA/TFA ratios.

**Title:** Supplementary Data 41.

**Description:** Distribution of PC/TFA and PUFA/TFA ratios in diabetic (n=3,100) and non-diabetic (n=27,169) individuals (NMR metabolomics, UK Biobank data).

**Title:** Supplementary Data 42.

**Description:** Distribution of MUFA/TFA, PC/TFA, and PUFA/TFA ratios across individuals reporting weight gain, no weight change, or weight loss compared with one year ago (NMR metabolomics, UK Biobank data).

**Title:** Supplementary Data 43.

**Description:** Distribution of the indicated fatty acids and specified FA ratios in diabetic (patients, n = 3,100) and non-diabetic (healthy, n = 27,169) individuals (NMR metabolomics, UK Biobank data).

**Title:** Supplementary Data 44.

**Description:** Spearman's correlation between selected metabolites and indicated health parameters is shown, based on NMR metabolomics data from the UK Biobank. CCI - Charlson Comorbidity Index.

**Title:** Supplementary Data 45.

**Description:** List of oligonucleotides used.

**Title:** Supplementary Data 46.

**Description:** Original outputs of the WormCat analysis performed in this study.
