# Supplementary material for: Aging-associated decline of phosphatidylcholine synthesis is a malleable trigger of natural mitochondrial aging
Source: Nat Commun. 2026 Apr 18;17:3589. doi: 10.1038/s41467-026-71508-7 (PMC13091796; doi:10.1038/s41467-026-71508-7)
Supplement: Supplementary file 49 — Reporting Summary [file 41467_2026_71508_MOESM49_ESM.pdf]

# nature portfolio

Corresponding author(s): Maria A. Ermolaeva

Last updated by author(s): Feb 26, 2026

# Reporting Summary

Nature Portfolio wishes to improve the reproducibility of the work that we publish. This form provides structure for consistency and transparency in reporting. For further information on Nature Portfolio policies, see our Editorial Policies and the Editorial Policy Checklist.

## Statistics

For all statistical analyses, confirm that the following items are present in the figure legend, table legend, main text, or Methods section.

| n/a | Confirmed | |
|---|---|---|
| ☐ | ☒ | The exact sample size (*n*) for each experimental group/condition, given as a discrete number and unit of measurement |
| ☐ | ☒ | A statement on whether measurements were taken from distinct samples or whether the same sample was measured repeatedly |
| ☐ | ☒ | The statistical test(s) used AND whether they are one- or two-sided<br>*Only common tests should be described solely by name; describe more complex techniques in the Methods section.* |
| ☐ | ☒ | A description of all covariates tested |
| ☐ | ☒ | A description of any assumptions or corrections, such as tests of normality and adjustment for multiple comparisons |
| ☐ | ☒ | A full description of the statistical parameters including central tendency (e.g. means) or other basic estimates (e.g. regression coefficient) AND variation (e.g. standard deviation) or associated estimates of uncertainty (e.g. confidence intervals) |
| ☐ | ☒ | For null hypothesis testing, the test statistic (e.g. *F*, *t*, *r*) with confidence intervals, effect sizes, degrees of freedom and *P* value noted<br>*Give P values as exact values whenever suitable.* |
| ☒ | ☐ | For Bayesian analysis, information on the choice of priors and Markov chain Monte Carlo settings |
| ☒ | ☐ | For hierarchical and complex designs, identification of the appropriate level for tests and full reporting of outcomes |
| ☐ | ☒ | Estimates of effect sizes (e.g. Cohen's *d*, Pearson's *r*), indicating how they were calculated |

*Our web collection on statistics for biologists contains articles on many of the points above.*

## Software and code

Policy information about availability of computer code

| | |
|---|---|
| Data collection | Data collection was performed as previously described (Espada et al, Nature Metabolism, 2020) |
| Data analysis | Data analysis was mostly performed as previously described (Espada et al, Nature Metabolism, 2020). In addition, WormCat 2.0 pathway analysis tool was used to analyze proteomics data, and specific code was used to analyze human gene expression and metabolomics data as stated in the main text. Code for the PEMT and UK Biobank analyses are available at https://doi.org/10.5281/zenodo.18554287 and https://github.com/donertas-group/mt-aging. Code for GTEx data preprocessing is available at https://github.com/mdonertas/aging_in_GTEx_v8 and https://doi.org/10.5281/zenodo.18554414. |

For manuscripts utilizing custom algorithms or software that are central to the research but not yet described in published literature, software must be made available to editors and reviewers. We strongly encourage code deposition in a community repository (e.g. GitHub). See the Nature Portfolio guidelines for submitting code & software for further information.

## Data

Policy information about availability of data

All manuscripts must include a data availability statement. This statement should provide the following information, where applicable:

- Accession codes, unique identifiers, or web links for publicly available datasets
- A description of any restrictions on data availability
- For clinical datasets or third party data, please ensure that the statement adheres to our policy

The mass spectrometry proteomics data have been deposited to the ProteomeXchange Consortium (Deutsch et al., 2020) via the PRIDE (Perez-Riverol et al., 2019)

partner repository available at http://www.ebi.ac.uk/pride, with the dataset identifier PXD024180. All code and data for the PEMT and UK Biobank analyses are available at https://doi.org/10.5281/zenodo.18554287 and https://github.com/donertas-group/mt-aging. Code for GTEx data preprocessing is available at https://github.com/mdonertas/aging_in_GTEx_v8 and https://doi.org/10.5281/zenodo.18554414. The mass spectrometric lipidomics data generated in this study have been deposited in the Metabolomics Workbench database (an international repository for metabolomics data and metadata, metabolite standards, protocols, tutorials and training, and analysis tools85) under the accession code PR002921 (ST004627; https://doi.org/http://dx.doi.org/10.21228/M83K12). Source data are provided with this paper.

## Research involving human participants, their data, or biological material

Policy information about studies with human participants or human data. See also policy information about sex, gender (identity/presentation), and sexual orientation and race, ethnicity and racism.

| | |
|---|---|
| Reporting on sex and gender | We utilized UK Biobank data field 31 for the sex information, which is acquired from central registry at recruitment. When analyses were conducted separately for females and males, the exact number of participants in each group is detailed in the manuscript/figure/figure legends. It is important to note that participant counts may differ slightly across analyses due to random fluctuations in the recorded measures, and thus provided individually for different analyses. |
| Reporting on race, ethnicity, or other socially relevant groupings | The full dataset was used without stratification by ethnicity. The majority of participants in the UK Biobank are of British ancestry, which reflects the overall composition of the dataset. |
| Population characteristics | Each analysis included all individuals with the metabolomics and other relevant measures available, without any additional filtering. A supplementary figure is provided with the description of the age range of participants, stratified by sex, for those individuals who had metabolomics measurements. |
| Recruitment | Readers are directed to the UK Biobank study documentation for complete details regarding recruitment. It is important to emphasize that the UK Biobank represents a population cohort rather than a disease-specific cohort, and it is known to exhibit a healthy participant bias. |
| Ethics oversight | All participants provided informed consent for their data to be used in research, in accordance with UK Biobank protocols. Additionally, participants who withdrew their data after the initial collection were excluded from the analyses as required by these protocols. This study has been conducted using the UK Biobank Resource under application number 129228. |

Note that full information on the approval of the study protocol must also be provided in the manuscript.

# Field-specific reporting

Please select the one below that is the best fit for your research. If you are not sure, read the appropriate sections before making your selection.

☒ Life sciences ☐ Behavioural & social sciences ☐ Ecological, evolutionary & environmental sciences

For a reference copy of the document with all sections, see nature.com/documents/nr-reporting-summary-flat.pdf

# Life sciences study design

All studies must disclose on these points even when the disclosure is negative.

| | |
|---|---|
| Sample size | For the survival analysis of C. elegans 2 cohorts of 70 animals were used per condition to achieve n over 100 at the end of the test, accounting for the spontaneous loss of animals during the experiment (Petrascheck and Miller, Front Genet 2017). For proteomics and lipidomics analysis 700-800 animals were chosen as optimal sample size based on previous experience (Espada et al, Nat Metabolism, 2020). For microscopy experiments (mitochondrial stress and mitochondrial morphology tests) the animal numbers were chosen on the basis of previous respective publications (Espada et al, Nat Metabolism, 2020; Burkewitz et al, Cell 2016). Cell culture experiments were performed as previously described (Espada et al., Nature Metabolism, 2020), and Seahorse assays were conducted according to Sharifi et al. (Nature Communications, 2024). In both cases, sample size was determined as previously described in the respective studies. |
| Data exclusions | Censoring of C. elegans was applied according to standard procedures (Zhao et al, Nat Commun 2017), specifically contaminated plates, spontaneously ruptured animals, bag of worms phenotype, missing animals and animals damaged during handling were noted as censored. |
| Replication | Each presented experiment was repeated at least 3 times; only experiments which showed reproducible results were included into publication. Figures show either representative results or a summary of all trials, as specified in the respective legends. |
| Randomization | For life span experiments and microscopy tests in C. elegans the needed number of animals was randomly picked from larger populations subjected to RNAi and other treatments. For the lifespan analysis 2 cohorts of 70 worms were randomly picked. Each test group included two 60mm dishes containing 70 worms to account for potential plate-to-plate differences. For microscopy, 3 independent replicas of 20 animals were randomly picked and analyzed per condition to account for potential plate-to-plate differences. For proteomics and lipidomics analysis at least 700 worms per condition were randomly allocated to replica plates and maintained as independent cultures until sample collection. For cell culture tests frozen cell cultures were thawed and seeded on plates without bias for further treatment and analysis. |
| Blinding | Blinding was not applied, as the experiments were either inherently unbiased (e.g., omics analyses) or independently replicated by more than one researcher in most cases. |

# Reporting for specific materials, systems and methods

We require information from authors about some types of materials, experimental systems and methods used in many studies. Here, indicate whether each material, system or method listed is relevant to your study. If you are not sure if a list item applies to your research, read the appropriate section before selecting a response.

## Materials & experimental systems

| n/a | Involved in the study |
| --- | --- |
| ☒ | ☐ Antibodies |
| ☐ | ☒ Eukaryotic cell lines |
| ☒ | ☐ Palaeontology and archaeology |
| ☐ | ☒ Animals and other organisms |
| ☒ | ☐ Clinical data |
| ☒ | ☐ Dual use research of concern |
| ☒ | ☐ Plants |

## Methods

| n/a | Involved in the study |
| --- | --- |
| ☒ | ☐ ChIP-seq |
| ☒ | ☐ Flow cytometry |
| ☒ | ☐ MRI-based neuroimaging |

# Eukaryotic cell lines

Policy information about cell lines and Sex and Gender in Research

| | |
| --- | --- |
| Cell line source(s) | BJ human foreskin fibroblasts were purchased from ATCC (Reference number CRL-2522) |
| Authentication | BJ cell line was authenticated and tested by ATCC using morphology, karyotyping and PCR based approaches. Link: https://www.lgcstandards-atcc.org/CellAuthenticationMatters |
| Mycoplasma contamination | BJ cell line was proved free from mycoplasma contamination by Mycoplasma PCR ELISA (Sigma Aldrich) |
| Commonly misidentified lines (See ICLAC register) | No misidentified cell lines were used in this study |

# Animals and other research organisms

Policy information about studies involving animals; ARRIVE guidelines recommended for reporting animal research, and Sex and Gender in Research

| | |
| --- | --- |
| Laboratory animals | Age-synchronized wild type and mutant C. elegans hermaphrodites were used in our tests; all nematode strains were obtained from the Caenorhabditis Genetics Center. The complete list of tested strains is provided in the Materials and Methods section of the manuscript. Animals were handled by using established methods and guidelines accepted in the field of C. elegans aging research. |
| Wild animals | Wild animals were not used in this study |
| Reporting on sex | Hermaphrodite animals were used in all C. elegans tests. |
| Field-collected samples | Field-collected samples were not used in this study |
| Ethics oversight | This study didn't involve materials or animal models which require ethical approval |

Note that full information on the approval of the study protocol must also be provided in the manuscript.

# Plants

| | |
| --- | --- |
| Seed stocks | NA |
| Novel plant genotypes | NA |
| Authentication | NA |
